# Knowledge, Attitude and Practices of Vector-Borne Disease Prevention during the Emergence of a New Arbovirus: Implications for the Control of Chikungunya Virus in French Guiana

**DOI:** 10.1371/journal.pntd.0005081

**Published:** 2016-11-01

**Authors:** Camille Fritzell, Jocelyn Raude, Antoine Adde, Isabelle Dusfour, Philippe Quenel, Claude Flamand

**Affiliations:** 1 Unité d’épidémiologie, Institut Pasteur de la Guyane, Cayenne, Guyane; 2 UMR “Emergence des Pathologies Virales” (Aix-Marseille University, IRD 190, INSERM 1207, EHESP), Marseille, France; UMR “Processus Infectieux en Milieu Insulaire Tropical” (INSERM 1187, CNRS 9192, IRD 249, Université de La Réunion), Réunion, France; 3 Unité d’entomologie médicale, Institut Pasteur de la Guyane, Cayenne, Guyane; 4 Inserm UMR 1085-IRSET Institut de recherche sur la santé, l’environnement et le travail, EHESP, Rennes, France; Common Heritage Foundation, NIGERIA

## Abstract

**Background:**

During the last decade, French Guiana has been affected by major dengue fever outbreaks. Although this arbovirus has been a focus of many awareness campaigns, very little information is available about beliefs, attitudes and behaviors regarding vector-borne diseases among the population of French Guiana. During the first outbreak of the chikungunya virus, a quantitative survey was conducted among high school students to study experiences, practices and perceptions related to mosquito-borne diseases and to identify socio-demographic, cognitive and environmental factors that could be associated with the engagement in protective behaviors.

**Methodology/Principal Findings:**

A cross-sectional survey was administered in May 2014, with a total of 1462 students interviewed. Classrooms were randomly selected using a two-stage selection procedure with cluster samples. A multiple correspondence analysis (MCA) associated with a hierarchical cluster analysis and with an ordinal logistic regression was performed. Chikungunya was less understood and perceived as a more dreadful disease than dengue fever. The analysis identified three groups of individual protection levels against mosquito-borne diseases: “low” (30%), “moderate” (42%) and “high” (28%)”. Protective health behaviors were found to be performed more frequently among students who were female, had a parent with a higher educational status, lived in an individual house, and had a better understanding of the disease.

**Conclusions/Significance:**

This study allowed us to estimate the level of protective practices against vector-borne diseases among students after the emergence of a new arbovirus. These results revealed that the adoption of protective behaviors is a multi-factorial process that depends on both sociocultural and cognitive factors. These findings may help public health authorities to strengthen communication and outreach strategies, thereby increasing the adoption of protective health behaviors, particularly in high-risk populations.

## Author Summary

Although dengue fever has been a focus of many awareness campaigns in Latin America, very little information is available about beliefs, attitudes and behaviors regarding vector-borne diseases among the population of French Guiana. At the initial onset of the first chikungunya outbreak, a quantitative survey was conducted among 1462 high school students aiming to study experiences, practices and perceptions related to mosquito-borne diseases and to identify factors that could be associated with protective behaviors. Chikungunya was less understood and perceived as a more dreadful disease than dengue fever. Students were clustered in three different groups according to their level of protection: “low” (30%), “moderate” (42%) and “high” (28%). Protective health behaviors were found to be performed more frequently among students who were female, lived with a parent who had a higher educational status, lived in an individual house, and had a better understanding of the disease. The results revealed that the adoption of protective behaviors is a multi-factorial process that depends on both socio-economic and cognitive factors. These findings may help the public health authorities to strengthen their communication and outreach strategy, thereby increasing the adoption of protective health behaviors, particularly in endemic countries and high-risk populations.

## Introduction

Chikungunya fever is a re-emerging arboviral disease caused by chikungunya virus (CHIKV), an alphavirus transmitted to humans primarily via the bite of an infected mosquito (*Aedes* spp. mosquito) [1]. Clinical onset is abrupt with high fever, headache, back pain, rash, myalgia and arthralgia, and symptoms generally resolve within 7–10 days [2]. The illness is usually self-limiting and resolves with time. Nevertheless, acute or chronic complications (e.g., polyarthralgia) can occur [3,4]. There is no specific treatment for chikungunya, and no vaccine is currently available [2]. Since its first identification in the early 50s in Tanzania, the spread of CHIKV has been the cause of many large outbreaks in Africa, Asia, and the Pacific Islands, especially during the last decade [1,5]. Before December 2013, CHIKV transmission had never been documented in the Americas despite annually reported imported cases and the presence of the main vectors *Ae*. *albopictus* and *Ae*. *aegypti* [6–9]. In December 2013, autochthonous cases were detected in the French overseas territory of Saint Martin and led to the rapid spread and transmission of CHIKV in the Caribbean countries and the Americas, including French Guiana, within 9 months [10,11].

In French Guiana, where *Ae*. *aegypti* mosquito was responsible for several dengue fever outbreaks [12–14], the first autochthonous cases of chikungunya were reported in February 2014 [15]. By June 2014, the epidemiological situation had evolved to moderate autochthonous transmission, epidemic clusters and a localized transmission chain. In September 2014, the situation worsened with an increasing number of clusters in the region [16]. Since the introduction of CHIKV in the territory, health authorities have reactivated the dengue fever control vector plan, based on an integrated vector management strategy promoted by the World Health Organization (WHO) and applicable to all vector-borne diseases. This strategy includes different approaches combining an environmental management program aimed at reducing breeding sites, using insecticides safely, biological control using organisms that reduce target species, providing education, increasing public awareness and promoting personal protection [17]. The active and persistent participation of the individuals and communities is a key factor in the achievement and sustainability of vector control programs, as the punctual interventions are generally ineffective at preventing outbreaks of vector borne diseases [18]. One important target group for such programs is the young generation, who can become more easily involved in community-based vector-source reduction campaigns [19,20]. In addition, participation at the individual level, such as use of insect repellent, mosquito netting or elimination of the indoor breeding sites, may also play an important role. Although education campaigns have increased public awareness of health risks related to dengue fever, which has strongly affected French Guiana, especially since 2006, it remains unclear to what extent this knowledge is associated with better preventive practices. Notwithstanding, no study had been previously conducted in French Guiana about the knowledge, attitude and practices related to vector-borne diseases. Additionally, studies conducted on health and illness perceptions indicated that these perceptions can significantly change over time according to health events [21–24]. Therefore, it was opportune to evaluate beliefs and practices of disease prevention with the increasing risk of arbovirus in the Americas [25,26], and follow their evolution according to epidemiologic settings.

The increasing risk of arbovirus transmissions in French Guiana prompted the need to document beliefs and behaviors related to vector-borne diseases and to determine the extent that socio-demographic, cognitive and environmental factors are associated with adequate protective behaviors.

The aims of the study were to describe and explore experiences, practices and perceptions of a new health threat related to vector-borne diseases among students of French Guiana; identify the main factors that are associated with the practice of protective behaviors; and quantify those associations.

## Methods

### Setting

**Fig 1:**
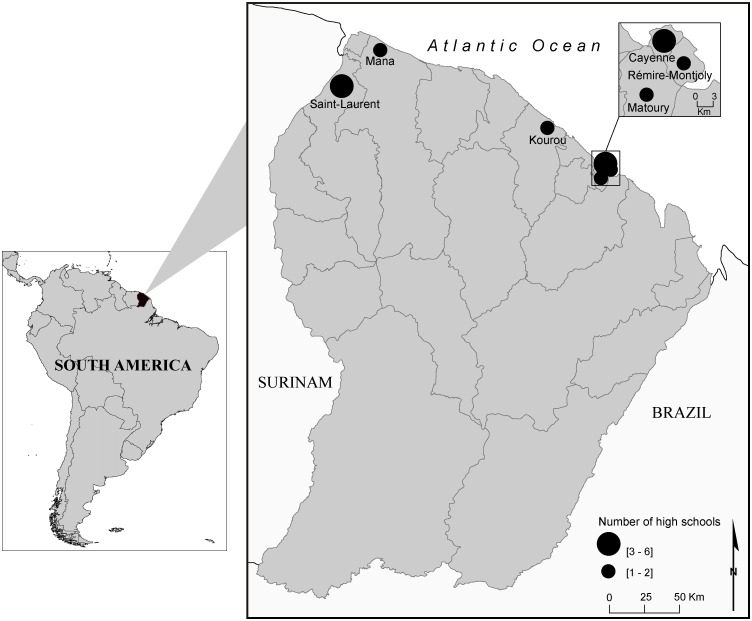
Spatial distribution of high schools in French Guiana, 2014.

French Guiana is a French overseas department located on the north-eastern coast of South America between Brazil and Surinam. At the time of the 2012 census, the population of the department was estimated at 239,500 individuals. This region is characterized by an extremely high birth rate and a high proportion of youth: 44% are under 20 years-old and only 4% are more than 65 years-old [27]. In 2014, the territory had 15 high schools localized on the coastline (Fig 1) where 12,400 students were registered (approximately 5.2% of the population). These students represented the target population of the study.

### Participants and procedures

A cross-sectional survey about “beliefs, attitudes and practices” among students of French Guiana was conducted from May 12–21, 2014, based on a random cluster, stratified sample of French Guiana students, with the aim of investigating 1,600 students using a 2-stage selection procedure to have a 95% confidence level associated to a 2.5% precision. The population was first divided into two strata: professional high schools and general high schools. Between those two strata, 77 classrooms (primary sampling unit) were selected from a simple random sampling. All of the individuals were investigated in every selected classroom using standardized self-questionnaires administered by local high school nurses.

The data were collected according to the rules established by the National Data Protection Authority (CNIL Declaration N°aaH1243499A), which is responsible for the ethical issues and protection of individual data collected in France. All of the information was collected anonymously, and an information letter was sent to the parent through the student’s liaison notebook informing them about the right to oppose the survey and to access the data. A signature was requested and checked by the school nurse before the survey begins.

### Questionnaire and measures

Aside from socio-demographic variables, collected data were grouped into five general categories: 1) environmental variables and exposure, 2) beliefs about disease, 3) perception of the risk associated with infectious diseases, 4) perception of the effectiveness of protective measures and 5) self-reported protective behaviors.

### Environmental and exposure variables

The questionnaire contained a wide range of items such as the type of housing, the presence or absence of potential breeding sites and potential factors associated with breeding sites, such as farming. In addition, respondents were asked how frequently they were bitten by mosquitoes (response options: ‘Never’, ‘Seldom’, ‘Sometimes’ and ‘Often’). Participants were also asked if they had ever seen or heard about “*Aedes* mosquitoes”, how frequently they practiced outdoor activities, and during what time of the day mosquito bites occurred. Participants were asked to report the occurrence of an acute febrile illness consistent with presumptive dengue virus and/or CHIKV infections.

### Cognitive and perceptual variables

A large range of beliefs were investigated; in particular, perceptions of the health threat, i.e., qualitative and quantitative judgments that individuals expressed when asked to evaluate a specific illness and the risk of contracting it [28]. To characterize these perceptions within the population, questions were drawn from the existing literature using the Brief Illness Perception Questionnaire (B-IPQ). This questionnaire measures five components: *the identity*- the symptom the patient associates with the illness; *the cause*- the personal ideas about etiology; *the time-line*- the perceived duration of the illness; *the consequences-* the expected effects and outcome; and *the treatment control*- the method of recovery from the illness [29]. Others questions were adapted from the methodological literature devoted to transmissible infectious diseases to complement the survey and assess perceived exposure, severity and susceptibility [30]. With the exception of the cause, the identity, the time-line and the mean of awareness, respondents answered each item by scoring them on a numeric scale of 0 to 10, with the meaning of each end-point indicated on the questionnaire.

Participants were asked to answer those questions in relation to dengue, chikungunya and three other infectious diseases occurring in French Guiana, malaria, human immunodeficiency virus (HIV) and yellow fever, by similarly giving a score of 0 to 10 for each question.

### Behavioral variables

For protective health behaviors, participants were asked how often they applied personal protective measures, such as wearing long-sleeved clothes or using repellent, and vector control measures, such as covering water receptacles. Then, participants were asked whether those behavioral recommendations were appropriate to prevent mosquito bites (response options: ‘Ineffective’, ‘A bit effective’, ‘Quite effective’, ‘Very effective’ and ‘Not sure’). Finally, respondents were asked to assess the constraint of each protective measure (response options: ‘Very constraining’, ‘Quite constraining’, ‘Bit constraining’ and ‘Not constraining’).

### Data analysis

Data were recorded using Access, and statistical analyses were performed using STATA12 software (Stata Corp., College Station, TX, USA) [31] and SPAD8 [32]. Primary sampling units and strata were taken into account to calculate estimations according to the design effect, and all estimations were obtained using STATA “svy” commands.

A multiple correspondence analysis (MCA) was used to display the relationships among the individual and structural factors, examining the association of protective behaviors with socio-economic and environmental factors. This method allows the simultaneous analysis of a large number of variables and their respective categories. The MCA method plots all the information represented by variables and individuals on a graph based on multiple factorial axes, and searches for patterns in the dataset, helping to identify the variables more closely associated with different groups. A matrix of eigenvalues was determined to identify a combination of variables that presented more stability in the factorial plan and explained the largest percentage of variability in the dataset. The number of dimensions was chosen by analyzing the decrease in eigenvalues. Variables were grouped as active or supplementary. Active variables were the respective frequency of each protection mean cited in the questionnaire with 4 modalities (never, seldom, sometimes and often) and the overall frequency of protection. Supplementary variables were the type of house, the high school sector, the parent’s level of education, the presence of a pool, an air-conditioning system, a yard and the name of the high school.

Following the MCA, a hierarchical cluster analysis was performed to determine the natural groupings of observations regarding protective behaviors. This cluster analysis encompassed a variety of mathematical methods for classifying groups of similar entities regarding the adoption of protective behaviors. We used a hierarchical agglomerative clustering algorithm that initially placed each individual in a separate cluster and then iteratively joined the two most similar clusters.

Finally, a logistic ordinal regression was performed to identify the differences among the socio-economic, environmental, exposure and cognitive variables associated with the level of protective behaviors. The clusters identified above were used as the dependent variable for the analysis. Factors that were determined significant in the univariate analysis were tested in a stepwise multivariate model as independent variables. The level of statistical significance was set to (P = 0.05).

## Results

In total, 1462 students from 13 high schools were included in this study, representing 12% of the whole student’s population. Two high schools were not surveyed in the study because of refusal from their director. All the students present at the time of the survey participated to the study. Respondents were aged 16.7 years old on average (range: 15–24).

The proportions of respondents who declared having or having had chikungunya and dengue were 0.8% (95% Confidence Interval (CI): 0.4–1.6) and 44.6% (95% CI: 41.5–47.6) respectively.

### Perceived threat associated with chikungunya virus

**Fig 2:**
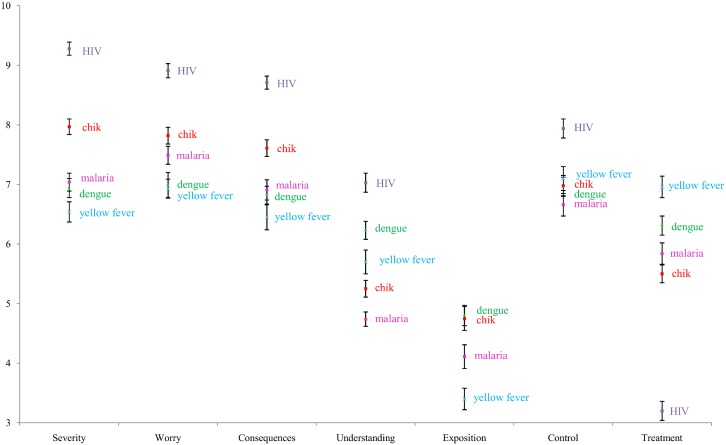
Mean threat perception scores regarding infectious diseases in French Guiana, 2014.

Mean threat perception scores are reported in Fig 2. We observed that chikungunya was a disease that worried more students than dengue fever. In fact, chikungunya displayed a higher mean score than dengue, malaria and yellow fever in relation to feeling worried, perceived severity and perceived consequences, although those scores remained lower than those associated with HIV. Students reported understanding less about chikungunya than dengue or yellow fever. Dengue and chikungunya displayed the same level of exposure, whereas students felt more exposed to chikungunya than malaria and yellow fever. In terms of control, students perceived chikungunya to be as avoidable as dengue and yellow fever, but more avoidable than malaria.

Students expressed the same perceived exposure for dengue (5.37, 95% CI: 5.12–5.63) and chikungunya (5.38, 95% CI: 5.14–5.63). However, regarding the time of illness, the perceived duration of chikungunya was longer than the duration of dengue, with 51% (95% CI: 47.3–54.1) of students thinking that chikungunya could last to three weeks to several months and only 32% (95% CI: 29.2–35.2) of students believing dengue fever had the same duration. Finally, the perceived level of information was higher regarding dengue (5.93, 95% CI: 5.69–6.14) than chikungunya (4.91, 95% CI: 4.50–5.31).

### Perceived threat associated with other infectious diseases

Malaria, which is primarily endemic to isolated and land areas, was reported with a higher mean score than dengue or yellow fever regarding feelings of fear and was less understood than dengue and chikungunya. HIV had the highest mean score for disease control, suggesting that students identified vector-borne disease protection more difficult than sexually transmitted disease protection. With the exception of perceived treatment efficacy, all HIV mean scores were higher than the midpoint value on the response scale adapted from the B-IPQ. Finally, in term of perceived treatment efficacy, yellow fever had the highest score among the reported values.

### Knowledge about vector-borne diseases

Participants identified different diseases that they believed to be transmitted by mosquitoes. More than half of the participants properly identified dengue (81%) and chikungunya (64%), more than a third properly identified yellow fever (40%) and malaria (36%), and 10% incorrectly identified HIV as transmitted by mosquitoes. Among students who correctly identified that mosquitoes could transmit dengue and chikungunya viruses, more than 80% could mention at least one symptom of these diseases. The most commonly mentioned symptom was tiredness (88%), followed by headache (82%). Overall, more than two-thirds of the participants reported that tiredness, headache, myalgia, arthralgia and skin rashes could be attributed to a mosquito-borne disease, in accordance with biomedical evidence on the clinical manifestation of these diseases.

Regarding the availability of a vaccine, 91% of the students reported that there was a vaccine against yellow fever, followed by 39% for a dengue vaccine, 34% for a malaria vaccine, and 16% for a chikungunya vaccine; 7% of the students thought that HIV was a vaccine-preventable disease.

Finally, when students were asked which method of awareness outreach they believed was the most appropriate for mosquito-borne diseases, school intervention, television and social networks were the most frequently cited (82, 76 and 74%, respectively).

### Beliefs and current protective behaviors related to mosquitoes

**Fig 3:**
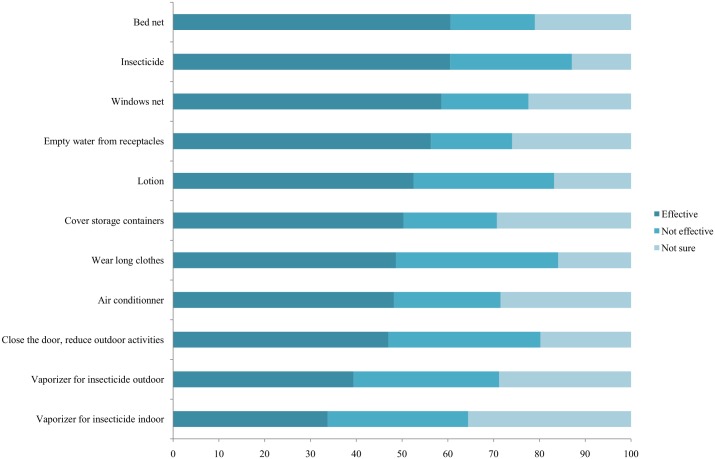
Perceived effectiveness of protective behaviors for reducing mosquito bites (%).

Although 77% of the student population was bitten by mosquitoes sometimes or often, only 54% of them reported taking preventive measures to reduce the number of mosquito bites. As shown in Fig 3, when asked about effectiveness of several preventive measures, the most commonly reported measures were bed nets (60.6%), followed by sprays (60.5%), window nets (58.6%) and removal of stagnant water from containers (58.5%). A perceived lack of effectiveness was expressed with regard to wearing protective clothing (a long-sleeved shirt and long trousers) (35%) and closing windows (33%).

**Fig 4:**
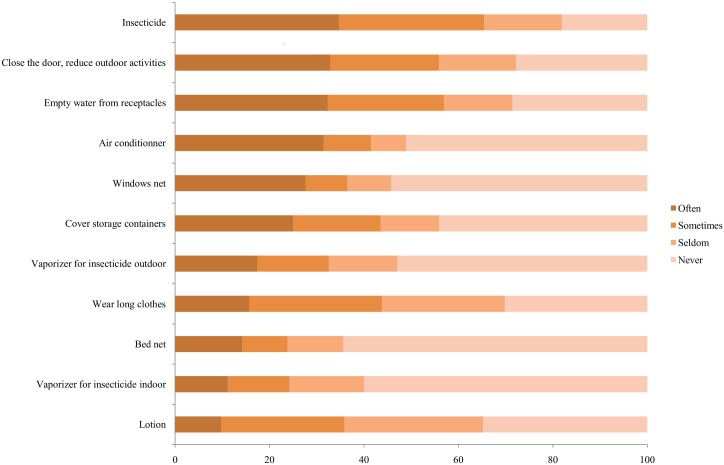
Self-reported protective behaviors aimed at reducing mosquito bites (%).

Self-reported behaviors to protect against mosquito bites are shown in Fig 4. The most frequently reported preventive measures were using indoor insecticide sprays (34.7%), closing windows (32.9%), removing stagnant water containers (32.3%) and using an air conditioner (31.5%). Surprisingly, when asked to note if one of the preventive measures was restrictive, students primarily noted insecticide sprays (54.4%). The most restricted option was the use of an indoor insecticide electric diffuser (67%) and the less constraining measure was the use of an air conditioner (26.1%). The most frequently expressed reasons for lack of protection were that students were not reminded to adopt protective behaviors (57%) and did not feel annoyed by mosquito bites (17%).

### Multiple correspondence analysis

**Fig 5:**
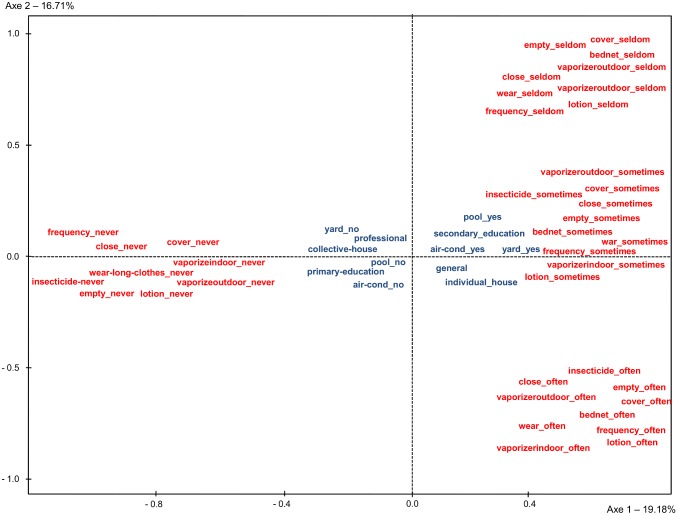
Multiple correspondence analysis plot for dimensions 1 and 2.

Graphic presentation of the variables, constructed in a series of 2-dimensional spaces is shown in Fig 5. This two-dimensional space enabled the mapping of protective behaviors as active variables and socio-economic as supplementary variables. Only the two first principal factors derived from the analysis were kept to plot the coordinates of the 62 variables. Factorial axis 1 captured 19.18% of the variability and distinguished individuals who adopted preventive measures from those who did not. The second axis captured 16.71% of the variability and showed a gradient in the level of protection. As shown in Fig 5, professional sector, collective housing, stay-at-home parent, parent with a low educational attainment, absence of yard or an air conditioner system were variables that were negatively associated with axis 1, as the response “never” was related to the frequency of several means of protection. Other variables that corresponded to a higher socio-economic status were positively associated with axis 1 as a positive attitude regarding protective behaviors. Finally, two high schools were negatively associated with axis 1.

**Fig 6:**
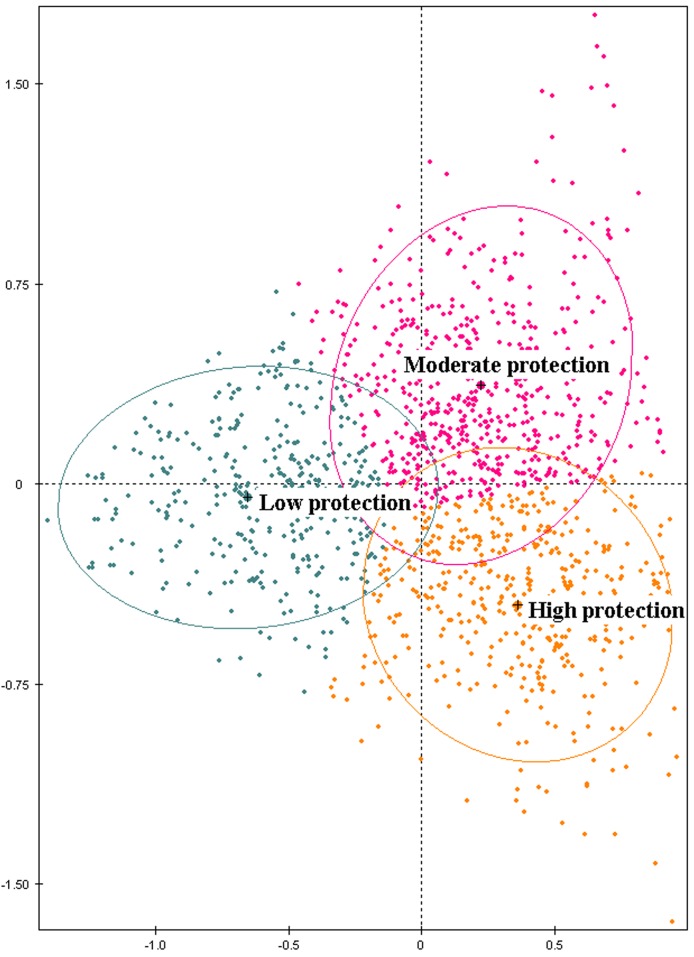
Typology of the population regarding the adoption of protective behaviors, factor 1 (19.18%) and factor 2 (16.71%).

The hierarchical cluster analysis identified three clusters related to the level of protection. Based on the usage patterns and the socio-economic and risk perception variables, this method offered a typology of the students as shown in Fig 6. The first cluster contained 450 individuals that never used protective means against mosquito bites. This category was characterized by the absence of a yard, a pool or an air conditioner system compared with the others clusters. Moreover, students registered in professional high schools and students with parents who had low educational level belonged to this cluster. The largest cluster accounted for 611 individuals (cluster 2) and concerned students that predominantly reported “seldom” or “sometimes” when asked about the frequency of usage protective measures. The last cluster, cluster 3, grouped together 401 individuals that reported “often” usage of protect measures. These last two clusters contained students with similar socio-economic patterns, such as the presence of a yard, a pool or an air conditioner system, parents with high educational level and registration in a general high school. Nonetheless, clusters could be distinguished with differences in risk perception variables. In cluster 3, students were very worried about chikungunya and dengue diseases, considering them very severe, but understandable diseases. Finally, in cluster 1, students primarily perceived a low efficacy related to several protective measures, whereas in cluster 3, students perceived these measures as very effective.

### Predictors of protective behaviors against mosquitoes

To identify socio-economic, environmental and individual factors associated with the level of self-reported protective behaviors, we performed univariate and multivariate ordinal regressions. Clusters resulting from the hierarchical cluster analysis were used as the dependent variables. The odds ratios (OR) resulting from the analysis and their significance are shown in Table 1. The results from the multivariate regression indicated different types of variables associated with the level of frequency of vector-borne disease protective behaviors. Among students, those who were female, lived with a parent that received an education higher than primary school, lived in an individual house and easily understood the diseases were more likely to report protective behaviors than other students.

**Table 1:** Logistic ordinal regression models predicting recommended protective behaviors.

Factors	Univariate Model		Multivariate Model	
	Unadjusted OR 95% CI	P-value	Adjusted OR 95% CI	P-value
**Dengue**	0.90 [0.62–1.30]	0.556		
**Chikungunya**	0.97 [0.35–2.64]	0.943		
**Gender**				
*Female*	referent		referent	
*Male*	0.69 [0.52–0.92]	0.017	0.73 [0.58–0.91]	0.008
**Sector**				
*General*	referent			
*Professional*	0.72 [0.53–0.99]	0.047		
**Level of parent education**				
*Primary school*	referent		referent	
*Some secondary school*	1.42 [0.81–2.47]	0.199	1.41 [0.82–2.42]	0.196
*Completed high school*	1.58 [1.01–2.47]	0.042	1.59 [1.12–2.26]	0.012
*Some college and higher*	1.46 [0.80–2.65]	0.197	1.30 [0.71–2.37]	0.371
**Household size**				
*[1–2]*	referent			
*[3–4]*	1.05 [0.80–1.36]	0.723		
*[5–6]*	0.84 [0.59–1.19]	0.321		
*7 and more*	1.48 [1.07–2.04]	0.018		
**Type of housing**				
*Collective*	referent		referent	
*Individual*	1.80 [1.15–2.80]	0.012	1.68 [1.09–2.61]	0.022
***Zone***				
*Rural*	referent			
*Half urban*	1.24 [0.90–1.72]	0.179		
*Urban*	0.87 [0.58–1.30]	0.493		
**Presence of yard**	1.50 [1.05–2.12]	0.025		
**Presence of pool**	1.14 [0.90–1.46]	0.251		
**Farming**	1.05 [0.74–1.50]	0.748		
**Frequency of mosquito bites**	0.86 [0.69–1.08]	0.187		
**Observation of Aedes**	1.21 [0.84–1.74]	0.27		
**Aedes knowledge**	0.23 [-0.08–0.56]	0.144		
**Perceived worry**				
*<4*	referent			
*[4–7]*	1.05 [0.79–1.40]	0.696		
*>7*	1.21 [0.72–2.03]	0.448		
**Perceived control**				
*<4*	referent			
*[4–7]*	1.25 [0.89–1.77]	0.175		
*>7*	1.22 |0.92–1.63]	0.15		
**Perceived severity**				
*<4*	referent			
*[4–7]*	1.20 [0.81–1.77]	0.332		
*>7*	1.46 [1.03–2.08]	0.034		
**Perceived consequences**				
*<4*	referent			
*[4–7]*	0.93 [0.61–1.42]	0.747		
*>7*	1.10 [0.80–1.50]	0.517		
**Perceived exposure**				
*<4*	referent			
*[4–7]*	1.32 [1.00–1.73]	0.047		
*>7*	1.26 [0.82–1.95]	0.267		
**Perceived treatment control**				
*<4*	referent			
*[4–7]*	0.99 [0.61–1.59]	0.966		
*>7*	1.09 [0.87–1.35]	0.403		
**Perceived cause**	0.97 [0.79–1.20]	0.820		
**Level of comprehension**				
*<4*	referent		referent	
*[4–7]*	1.66 [1.11–2.48]	0.016	1.73 [1.20–2.49]	0.005
*>7*	1.73 [1.19–2.52]	0.007	1.74 [1.24–2.43]	0.003
**Perceived level of information**	1.18 [0.90–1.56]	0.184		
**Perceived efficacy of several means**				
*Not effective*	referent			
*Effective*	1.68 [1.30–2.18]	<0.001		
*Very effective*	3.61 [2.46–5.30]	<0.001		

## Discussion

To our knowledge, this is the first published study on protective behaviors related to mosquito-borne diseases conducted in French Guiana. The survey was conducted during the emergence of chikungunya virus in French Guiana and nearly one year after the last dengue outbreak. Even if the student population was not representative of the entire population, the high school environment provided us an opportunity to conduct a large survey in the aftermath of the first reported confirmed cases of chikungunya in the region within an accessible and diversified population in terms of socioeconomic status. Additionally, the study represented an opportunity to investigate a perceived prevalence of the disease a few weeks after detection of the first autochthonous transmission clusters in the territory.

The method used in this study measured the perceived risk of chikungunya compared to various other health risks facing the public in French Guiana. The study showed that, with the notable exception of HIV, chikungunya was associated with the highest scores in term of perceived severity, worry and consequences, which is not surprising in a context of an emergence of a new virus. Our findings highlight that students in French Guiana were concerned and aware of the characteristics about vector-borne diseases. Approximately 81% and 64% of students knew that mosquitoes transmit dengue and chikungunya virus, respectively, and more than 80% were able to correctly mention at least one of the symptoms associated. Participants appeared to have good information about common symptoms and transmission. This awareness is likely attributed to a former infection of one of the mosquito-borne diseases or a result of a mass media campaign about dengue prevention. In fact, media coverage and the extent of reporting events may have exerted an influence on public perception of these diseases and therefore to produce positive changes in health-related behaviors [33,34].

It should be noted that among those who were bitten by mosquitoes, only half adopted protective behaviors. This result is somewhat unexpected since the perceived exposure to vectors (seeing bugs, being bitten) has been repeatedly found as one of the most important triggers for taking health protective actions [35,36]. This cannot be explained by an important perceived lack of behavioral control because this variable was not found to be associated with the adoption of preventive measures against mosquitoes. Nevertheless, we cannot exclude a sentiment of fatigue regarding the emerging infectious diseases and the continuous individual and collective efforts that are required to control them, given the growing number of public health warnings experienced by the local population over the past years. Moreover, many students expressed in additional comments that the means of protection were too much expensive and that they could not afford it. Several studies have explored cognitive factors and underlined the importance of public health beliefs in the adoption of effective preventive behaviors. Findings have notably shown that a lack of perceived behavioral control and a low perceived susceptibility to the threat inhibited sustained protective actions against vector-borne diseases; however, an increased understanding of the disease transmission led to better dengue prevention practices [37–41], and this factor has been importantly highlighted in our analysis.

Our findings hold important implications for the prevention of a new threat showing the importance for public health authorities to accommodate their strategies to rapidly strengthen the disease understanding in populations at risk.

The results showed that the adoption of protective behaviors is a multi-factorial process that depends both on socio-economic, environmental and cognitive factors. These factors should be further reviewed and considered in the development and implementation of future large-scale mosquito-borne disease prevention programs. In previously conducted infectious disease studies, these social and cognitive factors have consistently been found to influence the engagement in health protective behaviors [35,42,43]. However, in our study, only a few cognitive factors were associated with the protective health behaviors recommended by the public health authorities at the early stage of the epidemic. Unlike previous studies, this survey was conducted among students, thus, 80% of individuals were between 16 and 18 years old. This result can be explained by an age group effect in which the variability of cognitive variables was too low to have an impact on behaviors.

In contrast, a number of socioeconomic factors were found to shape the behavioral response to this emerging health threat. The multivariate model showed that girls with a parent who received education higher than primary school were more likely to report health protective behaviors than were other students. There was also an influence of the environmental variables on protective behaviors; people living in individual houses were more likely to have gardens and, therefore, were more likely to be exposed to mosquito bites during outdoor activities and likely required to take extra precautions to keep mosquitoes away. Finally, the results revealed that a moderate or high level of disease comprehension supported the adoption of protective health behaviors. This finding is rather surprising because many previous studies only revealed a weak association between health literacy and practices aiming to reduce the risk associated with vector borne diseases [36,44–46]

Last but not least, this study enabled us to identify two high schools associated with a high level of non-protective practices and provided to local education authorities the opportunity to target and modify health messages delivered in these institutions. Indeed, this finding highlighted also that school intervention was the best mode of awareness mentioned by the participants.

## Conclusions

The survey helped to characterize the public apprehension of several vector-borne diseases, as well as the nature and frequency of health protective behaviors among students to control and prevent their dissemination in the population. Given the importance of the public understanding of illnesses in the adoption of effective protective behaviors, this study shed light on the value of education campaigns aiming to improve the lay comprehension of the diseases. They may be a useful pre-requisite for the programs encouraging community participation in vector control.

## Supporting Information

S1 Checklist (DOCX)
